# Molecular Characterization and RNAi-Based Functional Validation of *Ache* in the Neonicotinoid Stress Response of the Melon Fly, *Zeugodacus cucurbitae*

**DOI:** 10.3390/insects17080767

**Published:** 2026-07-26

**Authors:** Momana Jamil, Shakil Ahmad, Hina Gul, Valeria Palma-Onetto, Yanping Luo

**Affiliations:** 1 School of Tropical Agriculture and Forestry, Hainan University, Haikou 570228, China; jamilmomana@gmail.com; 2 School of Ecology, Hainan University, Haikou 570228, China; 3 Guangdong Key Laboratory of Animal Conservation and Resource Utilization, Institute of Zoology, Guangdong Academy of Science, Guangzhou 510260, China; mshakil.aup@gmail.com; 4 State Key Laboratory for Quality and Safety of Agro-Products, Institute of Plant Protection and Microbiology, Zhejiang Academy of Agricultural Sciences, Hangzhou 310021, China; gulhina680@gmail.com; 5 Departamento de Química y Medio Ambiente, Universidad Técnica Federico Santa María, Avenida España 1680, Valparaíso 2390123, Chile; valeria.palmao@usm.cl

**Keywords:** *Zeugodacus cucurbitae*, acetylcholinesterase, neonicotinoids, cholinergic stress response, RNA interference, transgenerational effects, insecticide susceptibility

## Abstract

The melon fly, *Zeugodacus cucurbitae* (Coquillett), is a destructive tephritid pest of cucurbit crops, and neonicotinoids remain important tools for its management. Although neonicotinoids primarily target nicotinic acetylcholine receptors (nAChRs), their possible effects on acetylcholinesterase (AChE)-mediated cholinergic homeostasis in *Z. cucurbitae* remain poorly understood. In this study, we molecularly characterized the *Z. cucurbitae Ache* gene and evaluated its expression and functional contribution under dinotefuran, acetamiprid, and thiamethoxam stress. Bioinformatics analyses confirmed conserved cholinesterase features, including the FGESAG catalytic motif, catalytic triad, choline-binding site, oxyanion hole, and conserved disulfide-bond residues. Temporal and tissue-specific expression profiling showed high *Ache* transcript abundance in eggs, third-instar larvae, and adult heads. Larval exposure to LC_5_, LC_20_, and LC_50_ concentrations of the three neonicotinoids caused dose- and time-dependent mortality and significantly induced *Ache* expression, with dinotefuran producing the strongest transcriptional response. Parental exposure also impaired F_1_ fecundity, hatchability, larval survival, and adult emergence, with acetamiprid and thiamethoxam causing stronger transgenerational effects than dinotefuran. Oral RNAi significantly increased larval susceptibility upon re-exposure to neonicotinoids after 72 h. These findings indicate that *Ache* contributes to cholinergic stress response and susceptibility modulation in *Z. cucurbitae*, supporting its possible potential as an RNAi target for integrated pest management (IPM). However, practical utility at the field level requires further evaluation.

## Simple Summary

In this study, we characterized the acetylcholinesterase gene (*Ache*) in *Z. cucurbitae* and examined its response to three neonicotinoids: dinotefuran, acetamiprid, and thiamethoxam. Neonicotinoid exposure increased larval mortality and significantly induced *Ache* expression, showing the strongest acute toxic effect. Parental exposure also reduced fecundity, hatchability, larval survival, and adult emergence. Silencing *Ache* through oral RNA interference (RNAi) reduced gene expression and induced insecticide susceptibility. These findings identify *Ache* as a downstream stress-responsive gene that modulates neonicotinoid susceptibility under laboratory conditions.

## 1. Introduction

The melon fly, *Zeugodacus cucurbitae* (Coquillett) (Diptera: Tephritidae), is one of the most destructive fruit fly pests of cucurbit production systems in tropical and subtropical regions. Its broad host range, high reproductive capacity, internal larval feeding habit, and strong dispersal ability make this species difficult to manage using conventional control strategies alone [1,2]. Larval infestation reduces fruit quality through direct tissue destruction, microbial decay, premature fruit drop, and decreased market value, whereas adult populations create serious quarantine constraints for regional and international trade [3]. Because of these economic and phytosanitary impacts, *Z. cucurbitae* remains a major target for integrated pest management (IPM) programs, particularly in cucurbit-producing regions where repeated insecticide applications are still widely used [4,5].

Neonicotinoid insecticides, including dinotefuran, acetamiprid, and thiamethoxam, are important neuroactive compounds used against numerous agricultural pests [6,7]. These insecticides primarily act on insect nicotinic acetylcholine receptors (nAChRs), where they disrupt cholinergic neurotransmission and induce excitation, paralysis, and death [7,8,9]. Although their primary molecular targets are nAChRs, neonicotinoid exposure can also generate broader neurophysiological stress, including altered synaptic activity, changes in neurotransmitter turnover, oxidative imbalance, behavioral impairment, and reproductive disruption [10,11]. Sublethal exposure is especially relevant in pest management because insects surviving field-realistic or residual doses may show altered development, fecundity, offspring viability, and population growth potential [12,13]. Therefore, evaluating both lethal and sublethal outcomes is essential for understanding how neonicotinoids affect pest physiology beyond acute mortality.

Dinotefuran, acetamiprid, and thiamethoxam were selected because, although all three act primarily on insect nAChRs, they represent distinct neonicotinoid chemical groups and metabolic behaviors. Dinotefuran contains a tetrahydrofuranylmethyl nitroguanidine structure, acetamiprid is a chloropyridinyl cyanoamidine, and thiamethoxam is a chlorothiazolyl compound that can be metabolically converted to the active neonicotinoid clothianidin [14]. These differences may influence insecticide uptake, receptor interaction, metabolism, and toxicity. Comparing these compounds therefore allowed us to determine whether the *Ache* response represents a general response to neonicotinoids or varies among compounds with different structural and metabolic characteristics.

Acetylcholinesterase (AChE) is a key serine hydrolase that terminates cholinergic transmission by rapidly hydrolyzing acetylcholine in synaptic spaces. In insects, the *Ache* gene encodes a conserved cholinesterase enzyme containing characteristic structural features such as the FGESAG catalytic motif, catalytic triad, oxyanion hole, aromatic choline-binding gorge, and conserved cysteine residues required for proper folding and enzymatic stability [15]. In Cyclorrhaphan Diptera, comparative evolutionary studies indicate that the single functional *Ache* locus corresponds to the retained *Ache2* type lineage, which performs the principal synaptic AChE function in true flies [16,17,18]. Although AChE is the canonical target of organophosphate and carbamate insecticides, neonicotinoids act primarily on nAChRs and do not directly inhibit AChE. Nevertheless, cholinergic homeostasis depends on both receptor activation and the rapid hydrolysis of acetylcholine. Persistent stimulation of nicotinic receptors may therefore disturb synaptic balance and induce compensatory changes in *Ache* expression. Accordingly, *Ache* was investigated here as a neonicotinoid-responsive component of cholinergic stress regulation rather than as a direct neonicotinoid target. Thus, *Ache* is biologically relevant for investigating neurophysiological stress responses even when the insecticide class does not directly inhibit *Ache* [19].

In tephritid fruit flies, *Ache* has been extensively studied in relation to organophosphate resistance, target site insensitivity, and altered enzyme activity, particularly in species such as *Bactrocera dorsalis*, *Bactrocera oleae*, and *Ceratitis capitata* [20,21,22,23]. However, the molecular identity, expression dynamics, and functional contribution of *Ache* in *Z. cucurbitae* under neonicotinoid stress remain insufficiently characterized. This knowledge gap is important because transcriptional induction of neurophysiological genes after insecticide exposure may represent an adaptive or compensatory response that influences survival, susceptibility, and post-exposure fitness. Furthermore, RNA interference (RNAi) provides a powerful functional genomics approach for testing whether candidate genes contribute causally to insecticide stress outcomes, and oral dsRNA delivery has increasing relevance for species-specific pest management strategies in tephritids [24,25,26,27]. Although *Ache* responses have been reported in other insects, its molecular identity and functional contribution to neonicotinoid susceptibility have not been experimentally examined in *Z. cucurbitae*. The novelty of this study lies in integrating *Ache* characterization, exposure-responsive expression, offspring fitness assessment, and RNAi-based susceptibility testing in this species.

In the present study, we characterized the *Ache* gene of *Z. cucurbitae* and examined its role in the response to three neonicotinoid insecticides. Specifically, we analyzed conserved molecular features and phylogenetic placement of the predicted *Ache* protein; quantified developmental and tissue-specific *Ache* expression; evaluated larval mortality after exposure to LC_5_, LC_20_, and LC_50_ concentrations of dinotefuran, acetamiprid, and thiamethoxam; and determined the transcriptional response of *Ache* after insecticide exposure. We also assessed F_1_ generation effects following parental exposure and used oral RNAi to test whether *Ache* knockdown modifies larval susceptibility to neonicotinoid challenge. This integrated molecular, toxicological, and functional approach provides insight into the contribution of *Ache* to cholinergic stress response and susceptibility modulation in *Z. cucurbitae*.

## 2. Materials and Methods

### 2.1. Insect Colony and Rearing Conditions

A laboratory colony of the melon fly, *Z. cucurbitae*, was maintained at Hainan University, Haikou, China. The colony was established from field-collected individuals from Hainan Province and reared for three to four generations under controlled laboratory conditions at 27 ± 1 °C, 70 ± 5% relative humidity, and a 14 h light:10 h dark photoperiod [28]. Adult flies were maintained in insect-rearing cages and supplied a diet consisting of yeast hydrolysate and sugar at a ratio of 1:3 *w*/*w*, with water provided ad libitum. Eggs were collected and transferred to an artificial larval diet [29]. Larvae were reared on an artificial diet under the same environmental conditions until the required developmental stage. The 2nd instar larvae were used for insecticide exposure, RNAi, and post-RNAi bioassays unless otherwise stated.

### 2.2. Insecticides and Preparation of Stock Solutions

Technical grade dinotefuran (Hailir, Shandong, China), acetamiprid (Shanghai Lavaur Chemical Co., Ltd., Shanghai, China), and thiamethoxam (Shanghai Macklin Biochemical Technology Co., Ltd., Shanghai, China), each with 98% purity, were used in this study. Stock solutions were prepared separately at 100 mg L^−1^ in acetone. Working solutions were prepared using acetone: DMSO (1:1, *v*/*v*) and stored under dark conditions at 4 °C until use. For each treatment, 60 μL of working solution was mixed with 6 g of artificial diet, corresponding to 5 μL acetone and 5 μL DMSO g^−1^ diet [30]. Solvent controls without insecticide received the same solvent volume and composition as the insecticide treatments.

### 2.3. Larval Toxicity Bioassays and Lethal Concentration Determination

Larval toxicity was evaluated using a diet incorporation bioassay. For each insecticide, preliminary range finding assays were first conducted to identify concentration ranges producing approximately 5%, 20%, and 50% larval mortality. These preliminary assays were conducted with three biological replicates of 20 larvae each, and mortality was recorded after 24 h. Based on the estimated LC values, the concentrations corresponding to LC_5_, LC_20_, and LC_50_ used in subsequent experiments were 6.11, 16.09, and 44.36 mg/L for dinotefuran; 13.03, 33.44, and 89.79 mg/L for acetamiprid; and 12.32, 33.56, and 95.94 mg/L for thiamethoxam, respectively (Table S2). For each treatment, 60 μL of insecticide solution was thoroughly mixed into 6 g of artificial diet in subsequent experiments. Each concentration consisted of three independent biological replicates, with 30 larvae per replicate. Control larvae received a diet treated with the solvent mixture only. Larval mortality was recorded at 48 h and 72 h after exposure. Larvae were gently stimulated with a soft brush, and individuals showing no movement within 2 min were scored as dead.

### 2.4. Sample Collection After Neonicotinoid Exposure

To determine the transcriptional response of *Ache* under neonicotinoid stress, 2nd instar larvae were exposed to LC_5_, LC_20_, and LC_50_ concentrations of dinotefuran, acetamiprid, and thiamethoxam using the diet incorporation method described above. Surviving individuals were collected at 1, 3, 5, and 7 days after exposure. Only alive individuals were used for RNA extraction. Samples were immediately frozen in liquid nitrogen and stored at −80 °C. Each treatment and sampling time included three biological replicates. Solvent-treated larvae were collected at the same time point and used as controls.

### 2.5. Sample Collection of Developmental Stages and Adult Tissues

To determine developmental expression profiles, samples were collected from eggs, 1st instar larvae, 2nd instar larvae, 3rd instar larvae, pupae, newly emerged and 7-day-old adults. For tissue-specific expression analysis, 7-day-old adults were dissected on ice for head, midgut, ovary, and testis collection under a binocular stereomicroscope (Olympus SZX12, Tokyo, Japan). Each biological replicate consisted of pooled individuals or pooled tissues, depending on tissue size. All samples were immediately frozen in liquid nitrogen and stored at −80 °C until RNA extraction.

### 2.6. RNA Extraction and cDNA Synthesis

Total RNA was extracted from larvae, all developmental stages, and tissue samples using a TRI Reagent^®^ (Sigma-Aldrich, St. Louis, MO, USA according to the manufacturer’s instructions. RNA integrity was examined by 1% agarose gel electrophoresis, and RNA purity and concentration were measured using a NanoDrop 2000 spectrophotometer (Thermo Fisher Scientific, Waltham, MA, USA). First-strand cDNA was synthesized from 1 μg of total RNA using the PrimeScript reverse-transcription kit (Takara, Dalian, China) according to the manufacturer’s protocol. Synthesized cDNA was diluted to the appropriate working concentration using nuclease-free water and stored at −20 °C until use for PCR amplification and quantitative real-time PCR (qRT-PCR).

### 2.7. PCR Amplification and Cloning

The putative *Ache* sequence was identified from the available *Z. cucurbitae* genome assembly using homologous *Ache* sequences from closely related tephritid species as NCBI BLAST queries. Gene-specific primers were designed, and the full-length *Ache* fragment was amplified using PrimeSTAR Max DNA Polymerase (Takara, Dalian, China) with larval cDNA as the template.

PCR amplification was performed under the following conditions: 35 cycles of 95 °C for 30 s, 58 °C for 30 s, and 72 °C for 5 s/kb. PCR products were checked by 1% agarose gel electrophoresis. The expected amplicon was excised, purified, and ligated into a pMD18 T cloning vector (Takara, Dalian, China). Recombinant plasmids were transformed into *Escherichia coli* competent cells. Positive colonies were screened by colony PCR, and plasmid DNA was extracted using the Sangon Plasmid Miniprep Kit (Sangon Biotech, Shanghai, China).

### 2.8. Bioinformatic Characterization, Phylogenetic Analysis, and Structural Modeling of AChE

The confirmed *Ache* nucleotide sequence of *Z. cucurbitae* was translated into its deduced amino acid sequence using the ExPASy Translate tool (https://prosite.expasy.org/scanprosite/, accessed on 21 July 2026). The open reading frame and predicted protein length were verified using ORF finder (https://www.ncbi.nlm.nih.gov/orffinder/, accessed on 21 July 2026). Conserved cholinesterase residues were annotated by comparison with characterized insect AChE proteins and published cholinesterase active site information, including the FGESAG catalytic motif, the catalytic triad, oxyanion hole residues, choline-binding or catalytic anionic site residues, acyl-binding pocket residues, peripheral anionic site residues, and conserved cysteine residues.

For phylogenetic analysis, homologous AChE protein sequences from representative Diptera and other insect taxa were retrieved from the NCBI protein database using BLASTp tools (https://blast.ncbi.nlm.nih.gov/, accessed on 21 July 2026). The dataset included tephritid AChE sequences, Cyclorrhaphan, *Drosophila melanogaster* and representative mosquito Ache1 and Ache2 sequences to distinguish paralogous clades. Multiple sequence alignment was performed using MUSCLE implemented in MEGA 11. The best-fitting amino acid substitution model was selected in MEGA 11, and a maximum likelihood phylogenetic tree was reconstructed with 1000 bootstrap replicates. The final tree was visualized and annotated using Interactive Tree Of Life. The phylogenetic position of *Z. cucurbitae* AChE was interpreted according to the established evolutionary history of *Ache* genes in Diptera.

For three-dimensional structural analyses, the deduced *Z. cucurbitae* AChE amino acid sequence was submitted to SWISS MODEL for homology-based protein structure prediction. Where available, the model was also cross-checked against AlphaFold-predicted structures to confirm overall fold consistency. Template selection was based on sequence similarity, query coverage, global model quality estimation, and QMEAN score. The predicted model with the highest overall quality was selected for structural visualization. The model was used as a prediction-based representation of AChE architecture and not as an experimentally solved structure.

Structural conservation was evaluated by comparing the predicted *Z. cucurbitae* AChE model with a structurally characterized Dipteran AChE reference, *D. melanogaster* AChE, obtained from the Protein Data Bank. Structural superposition was performed in UCSF ChimeraX (https://www.cgl.ucsf.edu/chimerax/, accessed on 21 July 2026), by displaying the predicted AChE model as ribbon and surface representations. The catalytic core, active site gorge, and spatial arrangement of conserved functional residues were visually inspected after alignment. The catalytic triad residues Ser291, Glu420, and His533, the catalytic motif FGESAG, the choline-binding or catalytic anionic site residues Trp139, Tyr216, Tyr423, and Phe424, the acyl-binding pocket residues Trp324, Leu381, Phe383, and Phe424, and the peripheral anionic site residues Glu125, Tyr127, Met207, Trp374, and Tyr427 were mapped onto the predicted structure.

Functional residues were shown as stick representations and labeled according to their positions in the *Z. cucurbitae* AChE sequence. The active site gorge was displayed as a semitransparent molecular surface to illustrate the relative position of catalytic and substrate recognition residues. Final figure panels were arranged using Adobe Illustrator (version 30.2). The structural figure was used to support conserved AChE annotation, catalytic residue mapping, and fold conservation.

### 2.9. Quantitative Real-Time PCR Analysis

The qRT-PCR was used to measure *Ache* expression across developmental stages, adult tissues, insecticide exposure treatments, and RNAi treatments. qRT-PCR was performed using TB Green Premix Ex Taq II (Tli RNaseH Plus) (Takara, China) on an Applied Biosystems 7500 Real-Time PCR System. Each 10 μL qRT-PCR reaction contained 5 μL of TB Green Premix, 0.2 μL of ROX reference dye when required by the instrument platform, 3.5 μL of RNase-free water, 0.5 μL of diluted cDNA, and 0.4 μL each of forward and reverse primers at 10 μM. The amplification program was 95 °C for 30 s, followed by 40 cycles of 95 °C for 5 s and 60 °C for 30 s. A melting curve from 60 °C to 95 °C was performed to confirm primer specificity. Each sample was analyzed using three technical replicates and three independent biological replicates. *Actin* and *elongation factor 1 alpha* (*EFα1*) were used as endogenous reference genes for normalization [31]. Relative expression levels were calculated using the 2^−ΔΔCT^ method [32].

### 2.10. Synthesis of Double-Stranded RNA

Double-stranded RNA (dsRNA) targeting *Ache* and control ds*GFP* were synthesized using the T7 RiboMAX™ Express RNAi System (Promega, Madison, WI, USA) according to the manufacturer’s instructions. Gene-specific primers containing the T7 promoter sequence at the 5′ end were used to amplify the fragment of *Ache* and of *GFP* used as a negative control dsRNA. PCR amplification for dsRNA template preparation was performed under the following conditions: 95 °C for 3 min, followed by 35 cycles of 95 °C for 30 s, 58 °C for 30 s, and elongation time depending on fragment size at 72 °C, with a final extension at 72 °C for 10 min. PCR products were purified using the Universal DNA Purification Kit (TIAN GEN, Co., Ltd., Beijing, China). For in vitro transcription, the purified PCR product was used as a template in a 20 μL transcription reaction and incubated at 37 °C for 3 h. The reaction mixture was then treated with RNase and DNase solution for 30 min to remove single-stranded RNA and DNA templates.

The synthesized dsRNA was purified using sodium acetate and ethanol precipitation. Briefly, dsRNA was mixed with 3 M sodium acetate, pH 5.2, and 95% ethanol, incubated on ice for 5 min, and centrifuged at 13,000 rpm for 15 min at 4 °C. The supernatant was discarded, and the dsRNA pellet was washed with 70% ethanol, air-dried, and dissolved in nuclease-free water. dsRNA integrity was verified by 1% agarose gel electrophoresis, and concentration was measured using a NanoDrop 2000 spectrophotometer (Thermo Fisher Scientific, Wilmington, DE, USA). Purified dsRNA was diluted to the required working concentration and stored at −80 °C until use.

### 2.11. RNAi by Oral dsRNA Delivery

RNAi assays were conducted using early 2nd instar larvae. The artificial larval diet was supplemented with dsRNA by adding 60 μL of dsRNA solution to 6 g of diet and mixing thoroughly to ensure uniform distribution. Larvae were fed a diet containing ds*Ache* or ds*GFP* at a final dsRNA concentration of 1 μg μL^−1^. Control larvae were fed a diet containing ds*GFP* under identical conditions [28]. Each treatment consisted of three biological replicates, with 30 larvae per replicate. Larvae were allowed to feed a dsRNA-treated diet for 72 h, and the treated diet was replaced daily to maintain dsRNA availability. Two larvae from each replicate were collected at 24, 48, and 72 h after the start of dsRNA feeding to quantify *Ache* knockdown efficiency by qRT-PCR. Knockdown efficiency was calculated relative to the ds*GFP* control. Surviving larvae at 72 h after dsRNA feeding were used for subsequent neonicotinoid challenge assays.

### 2.12. Post-RNAi Neonicotinoid Challenge

To determine whether *Ache* knockdown affected larval susceptibility to neonicotinoids, larvae previously fed with ds*Ache* or ds*GFP* were exposed to dinotefuran, acetamiprid, or thiamethoxam using the diet incorporation method described above. For each insecticide, larvae were challenged with LC_5_, LC_20_, and LC_50_ concentrations. Each treatment consisted of three independent biological replicates, with 30 larvae per replicate. Larvae were maintained under standard rearing conditions, and mortality was recorded at 48 h after insecticide exposure. Individuals showing no movement after gentle stimulation with a soft brush for 2 min were recorded as dead. Mortality in ds*Ache*-treated larvae was compared with mortality in ds*GFP*-treated larvae to determine whether *Ache* silencing altered neonicotinoid susceptibility.

### 2.13. Assessment of Transgenerational Effects

To evaluate F_1_ generation effects of parental neonicotinoid exposure, surviving larvae from LC_5_, LC_20_, and LC_50_ exposure treatments were reared to adulthood under standard laboratory conditions. Newly emerged adults from each treatment were separated and paired for mating. Each treatment included 10 adult pairs, and each pair was recorded as an individual replicate.

Adults were provided with water and a yeast diet. Eggs were collected 10 days after adult emergence, when the flies had reached reproductive maturity, and were continued to be fed daily for 10 consecutive days using cucumber slices. Fecundity was recorded as the total number of eggs laid per female. Egg hatchability was calculated as the percentage of eggs that hatched successfully. F_1_ larval survival was calculated as the percentage of hatched larvae that survived to the third instar. Adult emergence was calculated as the percentage of F_1_ individuals that successfully emerged as adults. These parameters were used to assess parental exposure effects on offspring reproductive and developmental fitness.

### 2.14. Primer Information and Off-Target Assessment

All primers used for *Ache* cloning, qRT-PCR, ds*Ache* synthesis, and ds*GFP* synthesis are listed in Table 1. The ds*Ache* fragment used for dsRNA synthesis was 430 bp in length with a GC content of 47.7%. Potential off-target effects were assessed using BLASTn against the *Z. cucurbitae* reference genome (GCF_028554725.1) using default BLASTn (https://blast.ncbi.nlm.nih.gov/, accessed on 21 July 2026) parameters (word size = 11, E-value = 10), and no significant off-target gene matches were identified. The qRT-PCR primers were designed outside the dsRNA target region where possible to avoid amplification bias caused by residual dsRNA or degraded target fragments.

### 2.15. Statistical Analysis

All statistical analyses were performed using GraphPad Prism9 (GraphPad Software Inc., San Diego, CA, USA). Larval mortality data from concentration response assays were analyzed by probit regression to estimate LC_5_, LC_20_, and LC_50_ values with 95% confidence intervals. Gene expression data, knockdown efficiency, mortality after RNAi and insecticide challenge, and F_1_ fitness parameters were analyzed using one-way analysis of variance ANOVA. Treatment means were separated using Tukey’s honestly significant difference test. Data are presented as mean ± standard error. Differences were considered statistically significant at *p* < 0.05.

## 3. Results

### 3.1. Phylogenetic Placement and Conserved Sequence Features of AChE

**Figure 1:**
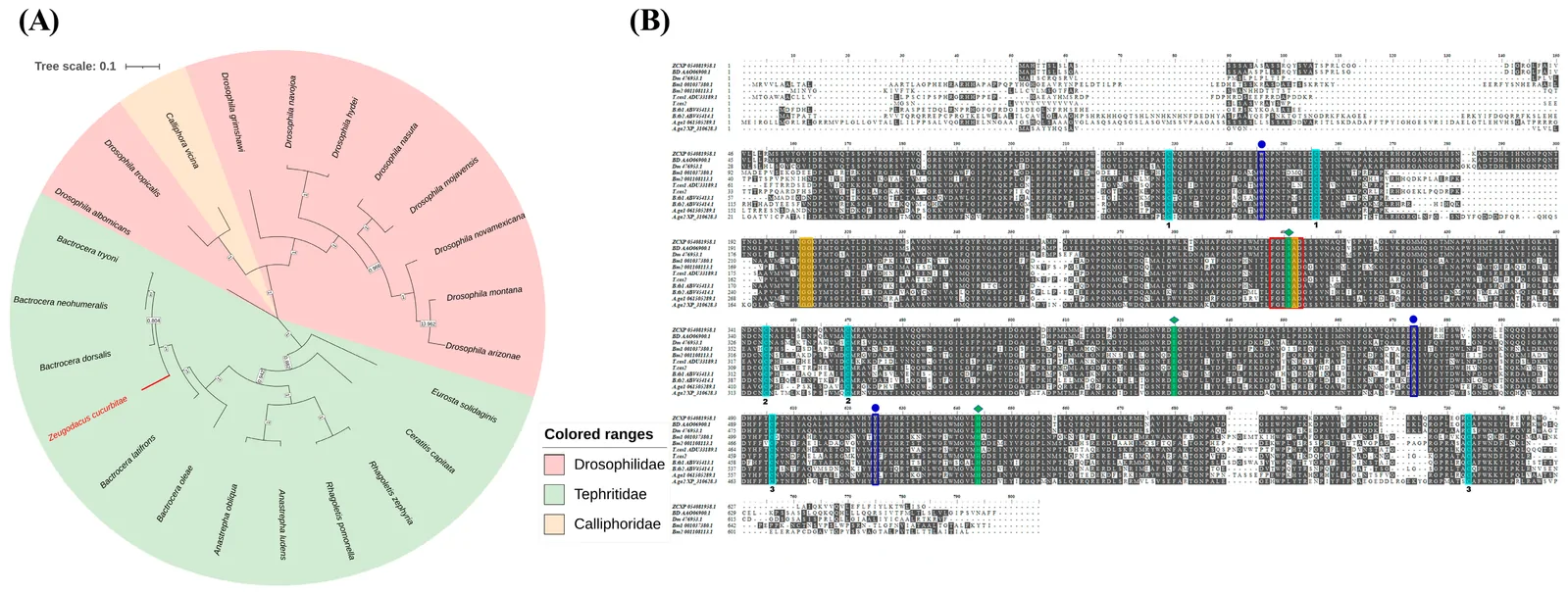
(**A**) Maximum likelihood phylogenetic tree of AChE protein sequences from representative Diptera. The *Z. cucurbitae* AChE sequence is highlighted in red. Bootstrap values are shown at major nodes, and the scale bar indicates amino acid substitutions per site. The clustering of *Z. cucurbitae* with tephritid AChE proteins supports its conserved relationship with Cyclorrhaphan acetylcholinesterases. (**B**) Multiple sequence alignment of AChE proteins showing conserved cholinesterase functional motifs. The conserved catalytic motif FGESAG is indicated in the red box; the solid diamond and green shade indicate the residues involved in the catalytic triad; the blue box with circles shows the choline-binding site; the oxianion hole is shown in the lime box; and the positionally conserved cysteine residues forming intramolecular disulfide bonds are in cyan with the numbers 1, 2, and 3. Conservation of these residues supports the annotation of the identified AChE sequence as a functional acetylcholinesterase.

Phylogenetic analysis of AChE proteins from representative Diptera placed the *Z. cucurbitae* AChE sequence within the Tephritidae clade, together with homologs from Bactrocera species and other higher flies. The topology separated Tephritidae from Drosophilidae and Calliphoridae, while the *Z. cucurbitae* sequence clustered closely with tephritid AChE proteins, supporting its conserved evolutionary relationship with Cyclorrhaphan AChE (Figure 1A). This placement is consistent with the interpretation that the *Z. cucurbitae* Ache corresponds to the Cyclorrhaphan Ache2 type lineage.

Multiple sequence alignment revealed strong conservation of functional cholinesterase motifs among Dipteran AChE proteins (Figure 1B). The *Z. cucurbitae* sequence contained the conserved catalytic motif FGESAG, the catalytic triad residues, choline-binding or catalytic anionic site residues, oxyanion hole residues, and conserved cysteine residues predicted to contribute to intramolecular disulfide bond formation. These conserved features indicate that the identified AChE sequence encodes a structurally conserved and catalytically competent AChE protein.

### 3.2. Predicted Structural Organization and Functional Residue Mapping of AChE

**Figure 2:**
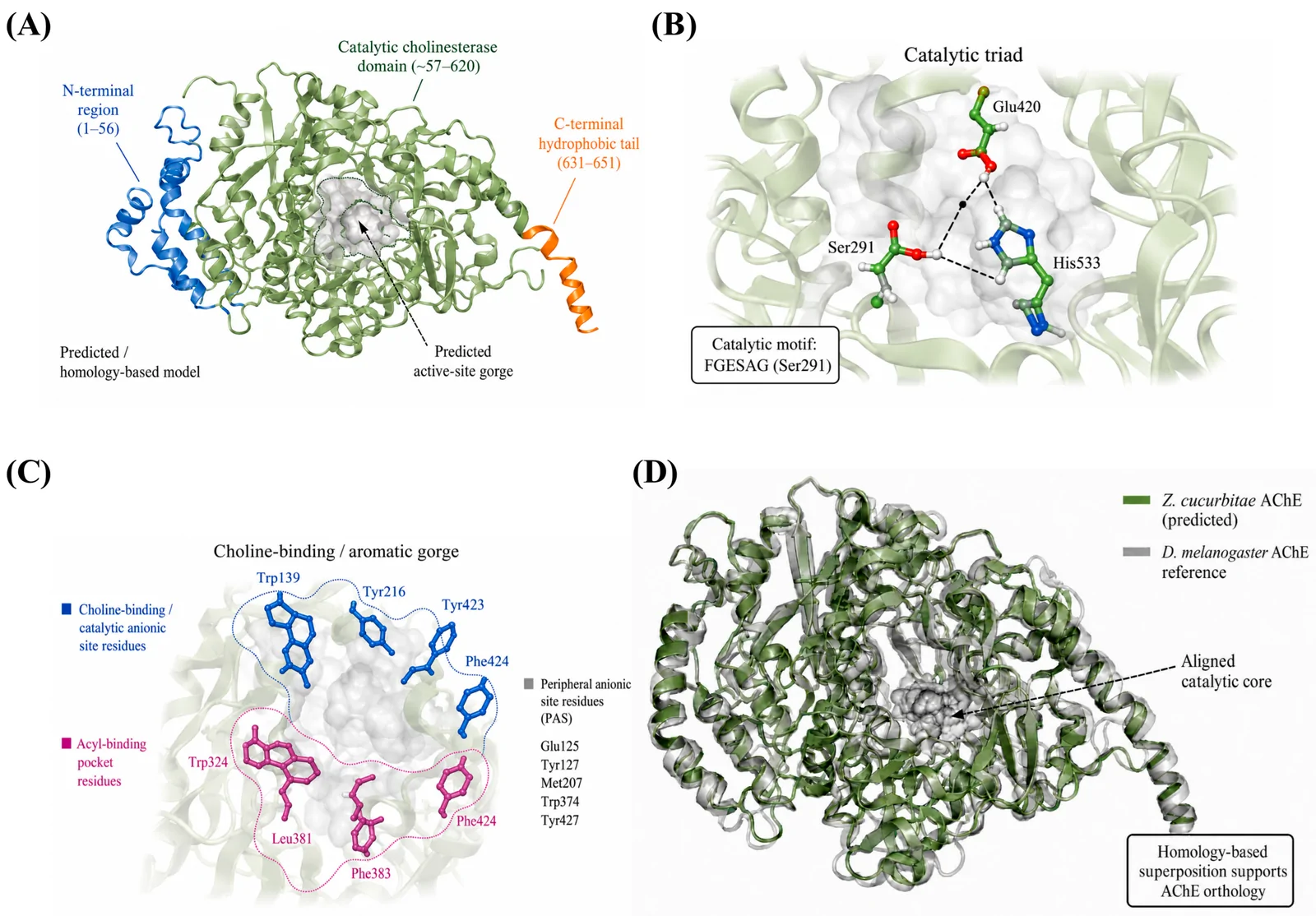
Predicted three-dimensional structure of *Z. cucurbitae* AChE and conserved functional residues. (**A**) Predicted or homology-based structural model of *Z. cucurbitae* AChE showing the N-terminal region, catalytic cholinesterase domain, C-terminal hydrophobic tail, and predicted active site gorge. (**B**) Enlarged view of the catalytic center showing the conserved catalytic triad Ser291, Glu420, and His533. Ser291 is located within the conserved FGESAG catalytic motif. (**C**) Structural mapping of residues forming the choline-binding or catalytic anionic site, acyl-binding pocket, and peripheral anionic site. The choline-binding or catalytic anionic site residues include Trp139, Tyr216, Tyr423, and Phe424. The acyl-binding pocket residues include Trp324, Leu381, Phe383, and Phe424. The peripheral anionic site residues include Glu125, Tyr127, Met207, Trp374, and Tyr427. (**D**) Structural superposition of the predicted *Z. cucurbitae* AChE model with a *D. melanogaster* AChE reference, showing conservation of the catalytic core and overall cholinesterase fold. The model is used to support functional residue mapping and conserved AChE architecture.

The predicted three-dimensional model of *Z. cucurbitae* AChE showed a typical cholinesterase fold, with an N-terminal region located at approximately amino acids 1 to 56, a central catalytic cholinesterase domain spanning approximately amino acids 57 to 620, and a C-terminal hydrophobic tail located at approximately amino acids 631 to 651 (Figure 2A). A predicted active site gorge was positioned within the catalytic domain, consistent with the architecture expected for insect AChE.

Mapping of conserved functional residues onto the predicted structure showed that the catalytic triad was spatially arranged within the active site gorge. The catalytic residues Ser291, Glu420, and His533 were positioned in proximity to one another, with Ser291 located within the conserved FGESAG catalytic motif (Figure 2B). The preservation of this catalytic arrangement supports the functional annotation of the *Z. cucurbitae* protein as an acetylcholinesterase.

The choline-binding or catalytic anionic site residues Trp139, Tyr216, Tyr423, and Phe424 were located along the aromatic gorge, whereas the acyl-binding pocket residues Trp324, Leu381, Phe383, and Phe424 occupied a neighboring region of the active site cavity (Figure 2C). Peripheral anionic site residues, including Glu125, Tyr127, Met207, Trp374, and Tyr427, were also mapped near the gorge entrance. Structural superposition with a *D. melanogaster* AChE reference showed alignment of the catalytic core, supporting conserved fold architecture and AChE orthology (Figure 2D).

### 3.3. Cloning of Ache and Developmental and Tissue-Specific Expression Profiles

**Figure 3:**
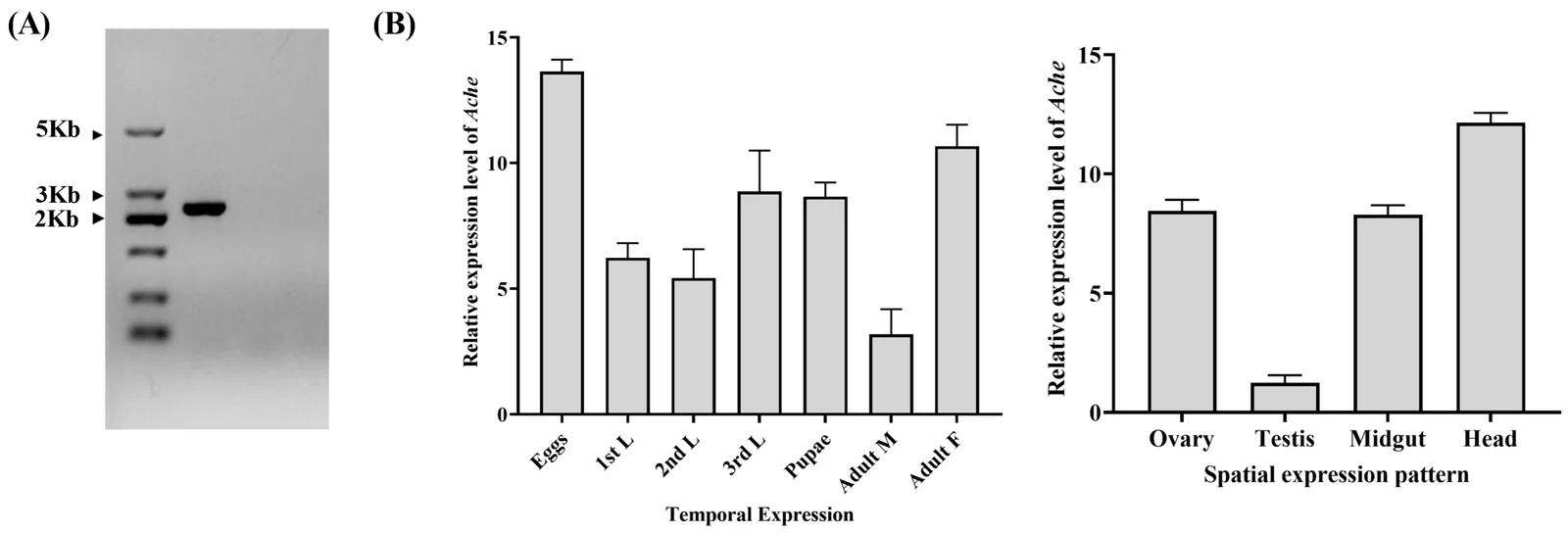
Amplification of *Ache* and expression profiles across developmental stages and adult tissues of *Z. cucurbitae*. (**A**) Agarose gel electrophoresis showing amplification of the expected size of the *Ache* fragment. (**B**) Relative expression of *Ache* across developmental stages and selected adult tissues, as determined by qRT-PCR. Developmental samples included eggs (*n* = 20), 1st L (*n* = 20), 2nd L (*n* = 10), 3rd L (*n* = 4), pupae (*n* = 3), adult M (*n* = 2), and adult F (*n* = 2). Tissue samples included head (*n* = 5), midgut (*n* = 15), ovary (*n* = 15), and testis (*n* = 15). Expression values were normalized to internal reference genes and are presented as mean ± SE from three biological replicates.

The *Ache* fragment was successfully amplified from *Z. cucurbitae* cDNA, producing a clear single band of the expected size of approximately 2.2 kb on agarose gel electrophoresis (Figure 3A). This result confirmed successful amplification of the target *Ache* transcript for subsequent sequence verification and functional analyses.

Developmental expression profiling showed that *Ache* transcript abundance varied across life stages (Figure 3B). The highest expression level was detected in eggs, followed by adult females (adult F), 3rd instar larvae (3rd L), and pupae. First (1st L) and second instar larvae (2nd L) showed moderate transcript abundance, whereas adult males (adult M) showed the lowest expression among the developmental stages examined. These results indicate that *Ache* expression is developmentally regulated and may be particularly important during embryonic development, late larval development, and female adult physiology.

Tissue-specific expression analysis revealed that *Ache* expression was highest in the head, followed by the ovary and midgut, while the lowest expression was detected in the testis (Figure 3B). The high expression in the head is consistent with the principal neurophysiological role of AChE in cholinergic synaptic transmission.

### 3.4. Neonicotinoid Toxicity and Post-Exposure Ache Expression

**Figure 4:**
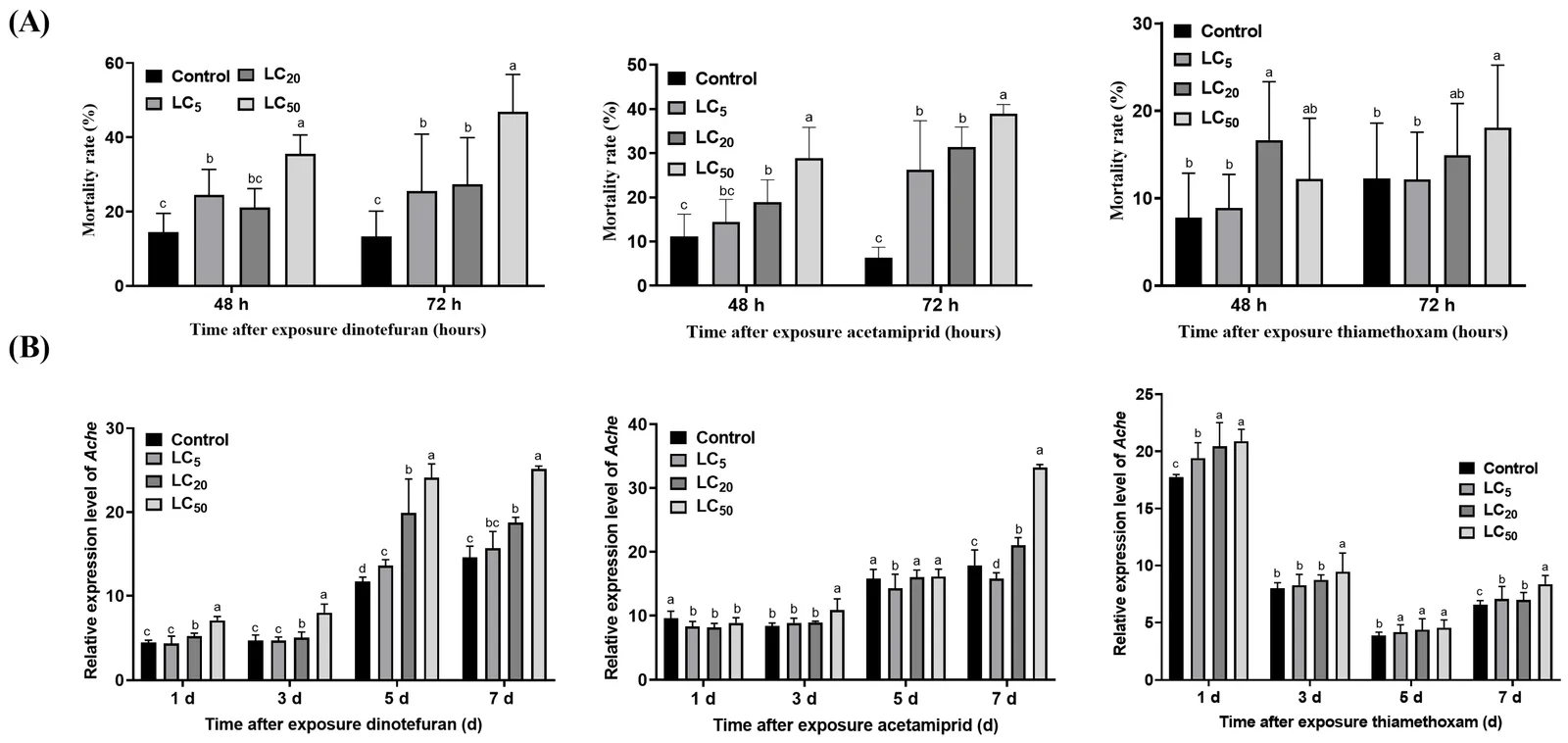
Larval toxicity of neonicotinoids and post-exposure *Ache* expression in *Z. cucurbitae*. For mortality assays, each treatment comprised three biological replicates of 30 larvae each. For expression analysis, each treatment comprised three independent biological replicates, with three technical qRT-PCR replicates per biological replicate. (**A**) Mortality after exposure to LC_5_, LC_20_, and LC_50_ concentrations of dinotefuran, acetamiprid, and thiamethoxam using a diet incorporation bioassay. Mortality was recorded at 48 h and 72 h after exposure. (**B**) Relative *Ache* expression in surviving larvae after exposure to LC_5_, LC_20_, and LC_50_ concentrations of the three neonicotinoids. Samples were collected at 1, 3, 5, and 7 days after exposure. Expression values were normalized to solvent-treated controls and internal reference genes. Data are presented as mean ± SE from three biological replicates. Different letters indicate significant differences among treatments or time points according to Tukey’s test at *p* < 0.05.

Larval diet incorporation bioassays showed concentration-dependent and time-dependent mortality after exposure to dinotefuran, acetamiprid, and thiamethoxam (Figure 4A). For dinotefuran, mortality increased with concentration at both 48 h and 72 h, reaching the highest level at LC_50_ after 72 h. Acetamiprid also caused a clear increase in mortality across LC_5_, LC_20_, and LC_50_ treatments, with higher mortality at 72 h than at 48 h. Thiamethoxam produced comparatively lower acute mortality, although mortality still increased at higher concentrations and later exposure time. Overall, dinotefuran produced the strongest acute larval toxicity, followed by acetamiprid, whereas thiamethoxam showed the lowest acute toxicity under the tested conditions.

Exposure to neonicotinoids significantly altered *Ache* transcript abundance in surviving larvae (Figure 4B). Dinotefuran induced a strong time-dependent and concentration-dependent increase in *Ache* expression. Expression remained relatively low during the first 3 days, increased markedly at day 5, and reached the highest level at day 7, especially in the LC_50_ treatment. Acetamiprid also induced *Ache* expression, with the strongest induction observed at day 7 under LC_50_ exposure. Thiamethoxam produced a different expression pattern, with relatively high transcript abundance at day 1 followed by reduced expression at days 3 and 5 and a moderate increase again at day 7. These results demonstrate that *Ache* is transcriptionally responsive to neonicotinoid stress, although the magnitude and temporal pattern of response differed among insecticides.

### 3.5. Transgenerational Effects of Parental Neonicotinoid Exposure

**Figure 5:**
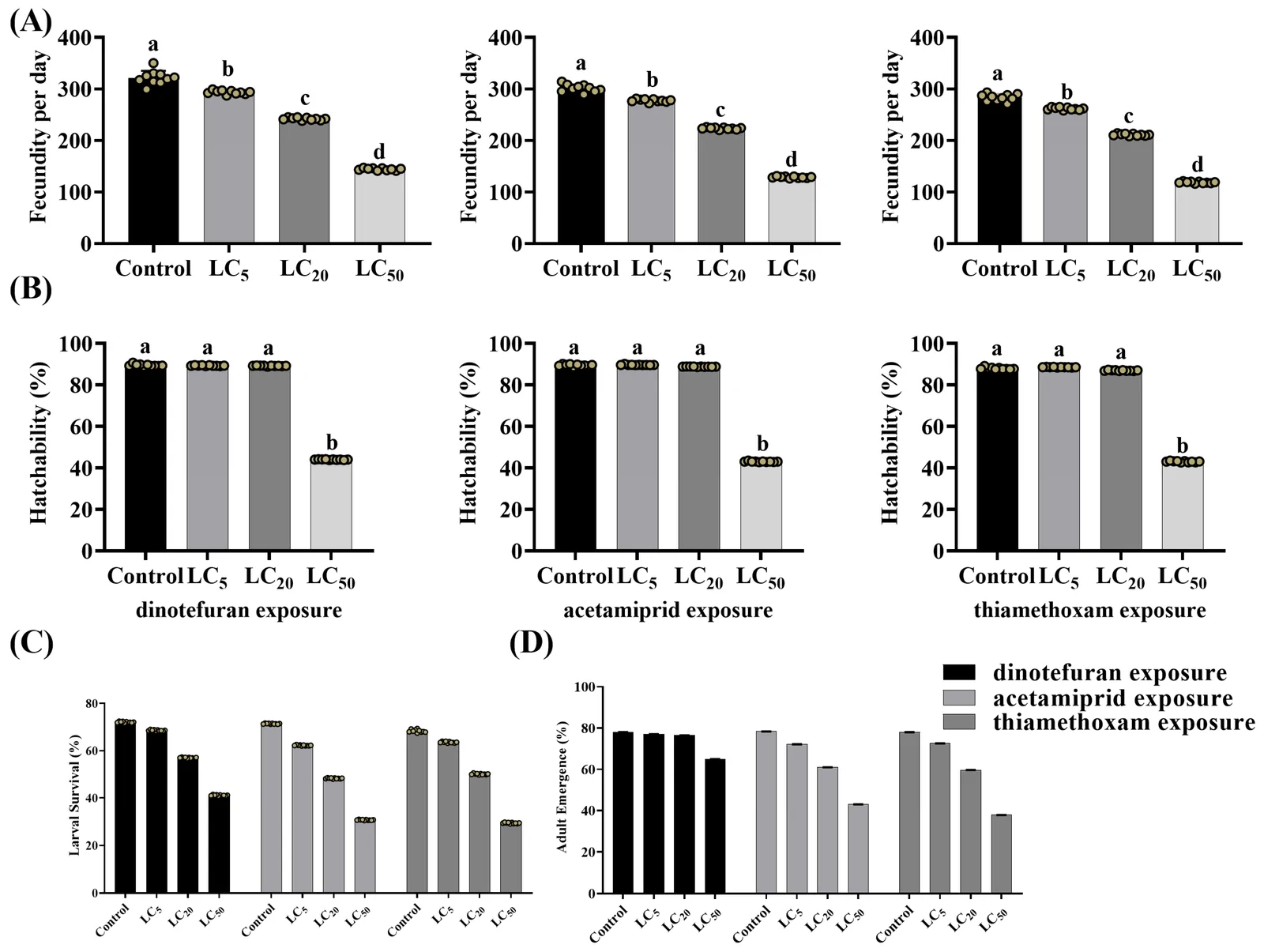
Transgenerational effects of parental neonicotinoid exposure on F_1_ reproductive and developmental traits of *Z. cucurbitae*. Parental insects exposed to LC_5_, LC_20_, and LC_50_ concentrations of dinotefuran, acetamiprid, and thiamethoxam were used to evaluate F_1_ generation performance. (**A**) Fecundity expressed as eggs per female per day. (**B**) Egg hatchability. (**C**) F_1_ larval survival. (**D**) F_1_ adult emergence. Values are presented as 10 individuals replicates, presented as yellow circles above the bars. Different letters above bars indicate significant differences among treatments within each insecticide according to Tukey’s test at *p* < 0.05. The results show that parental neonicotinoid exposure impaired offspring reproductive and developmental performance in a concentration-dependent manner.

Parental exposure to dinotefuran, acetamiprid, and thiamethoxam caused significant reductions in F_1_ reproductive and developmental performance (Figure 5). Fecundity decreased in a concentration-dependent manner after exposure to each insecticide (Figure 5A). In all three treatments, LC_50_ exposure produced the strongest reduction in eggs laid per female. Dinotefuran reduced fecundity from approximately 320 eggs per female per day in the control to approximately 140 eggs per female per day at LC_50_. Acetamiprid and thiamethoxam produced comparable or stronger reductions, with LC_50_ values close to approximately 130 and 120 eggs per female per day, respectively.

Egg hatchability remained high in control, LC_5_, and LC_20_ treatments but declined sharply at LC_50_ for all three insecticides (Figure 5B). Hatchability in the LC_50_ treatments decreased to approximately 43% to 45%, whereas lower concentrations showed limited reductions compared with the control. These results indicate that high parental neonicotinoid exposure strongly impaired embryonic viability in the F_1_ generation.

F_1_ larval survival also declined with increasing parental exposure concentration (Figure 5C). Dinotefuran caused a gradual reduction in larval survival, with the lowest survival observed at LC_50_. Acetamiprid and thiamethoxam produced stronger reductions, particularly at LC_20_ and LC_50_. Adult emergence followed a similar concentration-dependent trend (Figure 5D). Dinotefuran-treated groups retained comparatively higher adult emergence, whereas acetamiprid and thiamethoxam produced stronger reductions, especially at LC_50_. Together, these findings show that parental neonicotinoid exposure imposes measurable transgenerational fitness costs on *Z. cucurbitae*, affecting fecundity, hatchability, larval survival, and adult emergence.

### 3.6. RNAi-Mediated Ache Knockdown and Neonicotinoid Susceptibility

**Figure 6:**
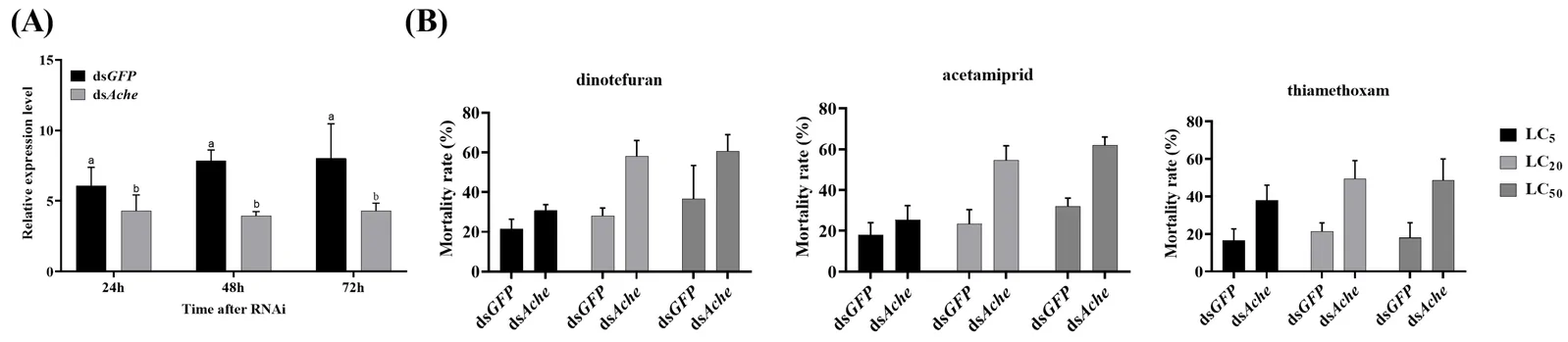
RNAi-mediated *Ache* knockdown and altered larval susceptibility to neonicotinoids in *Z. cucurbitae*. (**A**) Relative *Ache* transcript abundance in larvae after oral delivery of ds*Ache* for 24, 48, and 72 h. Larvae fed with ds*GFP* were used as negative controls. Expression values were determined by qRT-PCR and normalized to internal reference genes. (**B**) Mortality of ds*Ache* and ds*GFP*-treated larvae after subsequent exposure to dinotefuran, acetamiprid, and thiamethoxam. Post-RNAi insecticide challenge was performed using the same diet incorporation approach. Data are shown as mean ± SE from three biological replicates. Different letters indicate significant differences among treatments according to Tukey’s test at *p* < 0.05. Increased mortality in ds*Ache*-treated larvae indicates that *Ache* contributes to cholinergic homeostasis and susceptibility modulation under neonicotinoid stress.

Oral delivery of ds*Ache* effectively reduced *Ache* transcript abundance in larvae compared with the ds*GFP* control (Figure 6A). Knockdown was evident at 24 h and persisted through 48 h and 72 h after dsRNA feeding. The strongest silencing effect was observed at 72 h compared to the ds*GFP* control. These results confirm that oral dsRNA delivery can efficiently suppress *Ache* expression in *Z. cucurbitae* larvae.

Post-RNAi insecticide challenge showed that *Ache* knockdown increased larval susceptibility to dinotefuran, acetamiprid, and thiamethoxam (Figure 6B). In dinotefuran-treated larvae, mortality was consistently higher in the ds*Ache* group than in the ds*GFP* group across the tested exposure levels. A similar pattern was observed for acetamiprid, where ds*Ache*-treated larvae showed markedly higher mortality than ds*GFP* controls. Thiamethoxam exposure also produced higher mortality in *Ache* knockdown larvae compared with controls, particularly at higher exposure levels. These results indicate that reduced *Ache* expression compromises larval tolerance to neonicotinoid stress and supports a functional role for *Ache* in cholinergic homeostasis and susceptibility modulation in *Z. cucurbitae*.

## 4. Discussion

The present study integrates molecular annotation, toxicological response profiling, transgenerational assessment, and RNAi-based functional validation of *Ache* in the neonicotinoid stress response of *Z. cucurbitae*. Rather than identifying *Ache* as a direct neonicotinoid target, our findings support a more physiologically coherent model in which *Ache* contributes to cholinergic homeostasis and modulates the outcome of neonicotinoid exposure. This distinction is central because dinotefuran, acetamiprid, and thiamethoxam primarily act on insect nAChRs, whereas AChE is the canonical molecular target of organophosphates and carbamates [33,34]. Thus, the biological relevance of *Ache* in the present study lies in its capacity to regulate cholinergic disturbance after persistent nicotinic receptor stimulation, rather than in direct neonicotinoid detoxification or receptor-level resistance. We interpret *Ache* induction primarily as a downstream stress-associated response that may contribute to larval survival during neonicotinoid exposure. The increased mortality following *Ache* knockdown supports a protective contribution, but it does not establish a specific homeostatic pathway. *Ache* induction may also partly reflect a broader physiological stress or damage response. Similarly, the predicted structural model was used only for AChE annotation and conserved-residue mapping and does not provide evidence of neonicotinoid binding or AChE inhibition.

The induction of *Ache* expression after neonicotinoid exposure is consistent with a compensatory cholinergic response. However, increased transcript abundance does not necessarily indicate increased AChE protein abundance or enzyme activity. Future studies measuring AChE activity and acetylcholine levels are required to confirm this mechanism. Neonicotinoid activation of nAChRs can prolong excitatory signaling, disturb synaptic balance, and create physiological pressure on acetylcholine turnover [35]. Under such conditions, increased *Ache* transcription may represent an attempt to restore neurotransmitter homeostasis by enhancing acetylcholine hydrolysis capacity. Similar neonicotinoid-associated changes in *Ache* activity or expression have been reported in pollinating insects, where *Ache* was proposed as a biomarker of cholinergic disturbance after exposure to neonicotinoid-treated environments [36,37,38,39]. However, *Ache* responses are not uniform across insects and may vary according to dose, developmental stage, tissue, exposure route, and sampling time [40,41,42,43]. Therefore, the expression response observed in *Z. cucurbitae* is best interpreted as evidence of cholinergic stress responsiveness, not as proof that AChE is the primary toxicodynamic target of these compounds.

The contrasting acute and transgenerational effects of the three neonicotinoids are also biologically informative. Dinotefuran produced the strongest acute larval toxicity, whereas acetamiprid and thiamethoxam generated stronger impairment of several F_1_ traits. This divergence indicates that immediate mortality and delayed population-level consequences are not necessarily governed by the same physiological processes [43,44]. Acute mortality largely reflects rapid neurotoxicity, uptake, metabolic conversion, and detoxification kinetics in exposed larvae. The mechanisms underlying the observed F_1_ effects were not investigated in this study. Possible explanations include parental physiological stress, altered maternal provisioning, or persistent regulatory changes; however, these possibilities require experimental validation. Further studies are needed to determine the mechanisms linking parental neonicotinoid exposure with offspring performance. Sublethal and transgenerational effects of insecticides are increasingly recognized as critical determinants of pest population trajectories because they influence population renewal even when parental mortality is incomplete [45,46]. For pest management, this means that compounds with moderate acute toxicity may still produce important demographic suppression if they strongly impair reproduction or offspring viability.

The RNAi experiment provides the functional link between *Ache* expression and insecticide stress outcome. Oral ds*Ache* delivery substantially reduced transcript abundance and increased larval mortality after neonicotinoid challenge, indicating that normal *Ache* expression contributes to survival under cholinergic stress. However, this effect may reflect a general impairment of cholinergic homeostasis rather than a neonicotinoid-specific interaction. Testing *Ache*-silenced insects against other insecticide classes and non-insecticidal stressors would help distinguish between specific and general susceptibility effects. Mechanistically, reduced AChE levels may restrict the larva’s ability to hydrolyze acetylcholine efficiently, thereby intensifying synaptic imbalance during nAChR overstimulation. This interpretation is consistent with the central neurophysiological role of AChE in terminating cholinergic transmission [47,48]. It is also consistent with insect RNAi studies showing that *Ache* silencing can alter AChE activity, fitness, and insecticide susceptibility [49,50,51,52]. However, Hymenoptera and Diptera differ in their evolutionary history, *Ache* gene complement, expression patterns, and physiological responses to insecticides. Therefore, findings from bees should be considered supportive comparative evidence rather than direct mechanistic evidence for *Z. cucurbitae*. Nevertheless, the present effects should be described as susceptibility modulation rather than direct resistance reversal. In other insects, *Ache* silencing has more consistently enhanced susceptibility to organophosphates and carbamates than to neonicotinoids, which reinforces the need for cautious interpretation.

The transgenerational component strengthens the ecological relevance of this study. RNAi and insecticide studies often focus only on immediate mortality, but pest suppression depends on both survival and reproductive output. The observed reductions in F_1_ fecundity, hatchability, larval survival, and adult emergence indicate that neonicotinoid exposure can affect demographic traits that determine population growth. When interpreted together with the RNAi data, these findings suggest that interfering with cholinergic homeostasis may increase acute susceptibility, while neonicotinoid exposure itself can produce longer-term reproductive and developmental costs [53]. This dual perspective is useful for IPM because it links molecular stress response with population-relevant endpoints.

## 5. Conclusions

Overall, the evidence supports a model in which *Ache* is a conserved cholinergic response gene that may help maintain neurophysiological stability in *Z. cucurbitae* under neonicotinoid stress. Neonicotinoid exposure induces *Ache* expression, likely reflecting compensatory adjustment of acetylcholine turnover, while RNAi-mediated silencing of *Ache* compromises larval survival during subsequent insecticide challenge. This makes *Ache* a functionally relevant susceptibility modulator, but not the primary neonicotinoid target. By linking conserved AChE structure, inducible transcriptional response, RNAi-mediated functional validation, and F_1_ fitness costs, this study provides a biologically meaningful framework for incorporating cholinergic stress response genes into future integrated management strategies for *Z. cucurbitae*.

## Figures and Tables

**Table 1:** Primer sequences used in this study.

**Application**	**Primer Name**	**Sequence (5′–3′)**	**Product Size (bp)**
Ache cloning	Ache-F	CAGTCGTCAATCAGCTGTG	2247
	Ache-R	CTGACAAGTTGCTGTACTC	
qRT-PCR	qAche-F	CGGTCAGCCATTGAATAC	289
	qAche-R	CAGCACTTGTACCACCTTC	
dsRNA synthesis (Ache)	dsAche-F	GGATCCTAATACGACTCACTATAGGCAGCTGATTTACGTGTAC	430
	dsAche-R	GGATCCTAATACGACTCACTATAGGGTATTCAATGGCTGACCG	
dsRNA synthesis (control)	dsGFP-F	GGATCCTAATACGACTCACTATAGGGCAGTGGGAGGGTGAA	711
	dsGFP-R	GGATCCTAATACGACTCACTATAGGGTTGACGAGGGTGTCTC	
EFα1	qpcr-F	CGTTGGTGTCAACAAGATGG	230
	qpcr-R	TGCCTTCAGCATTACCTTCC	
Actin	qpcr-F	GACTCGTACGTCGGTGAC	200
	qpcr-R	CTGTGTCATCTTCTCACGG	

## Data Availability

The original contributions presented in this study are included in the Supplementary Material. Further inquiries can be directed to the corresponding authors.

## Supplementary Materials

The following supporting information can be downloaded at: https://www.mdpi.com/article/10.3390/insects17080767/s1, Table S1: Phylogenetic tree information; Table S2: Insecticide toxicity evaluation.
