# Supplementary material for: Molecular Characterization and RNAi-Based Functional Validation of Ache in the Neonicotinoid Stress Response of the Melon Fly, Zeugodacus cucurbitae
Source: Insects. 2026 Jul 26;17(8):767. doi: 10.3390/insects17080767 (PMC13513486; doi:10.3390/insects17080767)
Supplement: Supplementary file 1 [file insects-17-00767-s001.zip › insects-4428787-supplementary.pdf]

Table S1. All the species along with accession number used for phylogenetic tree construction. Tephritid AChE sequences were included to assess the phylogenetic placement of *Z. cucurbitae* within Tephritidae, whereas non-tephritid dipteran and other insect sequences were included to provide broader evolutionary context and to distinguish the Ache1 and Ache2 lineages.

Accession Number	Species
XM_054225983.1	*Zeugodacus cucurbitae*
XM_049449431.1	*Bactrocera dorsalis*
XR_007665922.1	*Bactrocera neohumeralis*
XM_036361577.2	*Bactrocera oleae*
XM_040099503.1	*Bactrocera tryoni*
XM_018931157.1	*Bactrocera latifrons*
XM_012301167.2	*Ceratitis capitata*
XM_054870657.1	*Anastrepha obliqua*
XM_036468618.1	*Rhagoletis pomonella*
XM_054090392.1	*Anastrepha ludens*
XM_017613991.1	*Rhagoletis zephyria*
XM_067762078.1	*Eurosta solidaginis*
XM_018016069.1	*Drosophila arizonae*
XM_032737321.1	*Drosophila grimshawi*
XM_064696528.1	*Drosophila montana*
XM_001998863.4	*Drosophila mojavensis*
XM_030702965.1	*Drosophila novamexicana*
XM_023307901.2	*Drosophila hydei*
XM_060794866.1	*Drosophila nasuta*
XM_030383478.1	*Drosophila navojoa*
XM_034261255.2	*Drosophila albomicans*
XM_065515739.1	*Calliphora vicina*
XM_015177081.3	*Drosophila willistoni*
XM_068301162.1	*Drosophila tropicalis*
PP178544.1	*Haemaphysalis longicornis*

Table S2. Toxicity of insecticides against *Zeugodacus cucurbitae*.

Insecticide	Slope ± SE	χ^2	df	P value	LC_5 (95% CI) (mg/L diet)	LC_{20} (95% CI) (mg/L diet)	LC_{50} (95% CI) (mg/L diet)
Dinotefuran	1.91 ± 0.21	6.16	3	0.104	6.11 (4.04–9.25)	16.09 (12.51–20.69)	44.36 (35.84–54.91)
Acetamiprid	1.96 ± 0.22	6.43	3	0.093	13.03 (8.64–19.64)	33.44 (26.13–42.79)	89.79 (73.02–110.41)
Thiamethoxam	1.85 ± 0.22	4.43	3	0.219	12.32 (7.94–19.10)	33.56 (25.94–43.43)	95.94 (76.74–119.93)

LC values were estimated by probit regression using $\log_{10}$-transformed insecticide concentrations. Each concentration consisted of three biological replicates with 20 larvae per replicate. Mortality was recorded after 24 h exposure. Control mortality remained ≤5%, and Abbott's correction was therefore not applied.
